# Design and Testing of a Bionic Dancing Prosthesis

**DOI:** 10.1371/journal.pone.0135148

**Published:** 2015-08-18

**Authors:** Elliott J. Rouse, Nathan C. Villagaray-Carski, Robert W. Emerson, Hugh M. Herr

**Affiliations:** 1 Biomechatronics Group, MIT Media Lab, Massachusetts Institute of Technology, Cambridge, Massachusetts, United States of America; 2 Department of Electrical Engineering and Computer Science, Massachusetts Institute of Technology, Cambridge, Massachusetts, United States of America; 3 A Step Ahead Prosthetics, Burlington, Massachusetts, United States of America; University of Manchester, UNITED KINGDOM

## Abstract

Traditionally, prosthetic leg research has focused on improving mobility for activities of daily living. Artistic expression such as dance, however, is not a common research topic and consequently prosthetic technology for dance has been severely limited for the disabled. This work focuses on investigating the ankle joint kinetics and kinematics during a Latin-American dance to provide unique motor options for disabled individuals beyond those of daily living. The objective of this study was to develop a control system for a bionic ankle prosthesis that outperforms conventional prostheses when dancing the rumba. The biomechanics of the ankle joint of a non-amputee, professional dancer were acquired for the development of the bionic control system. Subsequently, a professional dancer who received a traumatic transtibial amputation in April 2013 tested the bionic dance prosthesis and a conventional, passive prosthesis for comparison. The ability to provide similar torque-angle behavior of the biological ankle was assessed to quantify the biological realism of the prostheses. The bionic dancing prosthesis overlapped with 37 ± 6% of the non-amputee ankle torque and ankle angle data, compared to 26 ± 2% for the conventional, passive prosthesis, a statistically greater overlap (*p* = 0.01). This study lays the foundation for quantifying unique, expressive activity modes currently unavailable to individuals with disabilities. Future work will focus on an expansion of the methods and types of dance investigated in this work.

## Introduction

For decades, prosthetic design and control research has focused on enhancement of activities of daily living, such as ascending and descending stairs and inclines [1,2]. Such work has provided considerable advancement in community mobility and substantially impacts the quality of lives of these individuals. However, because prosthetic devices are designed for such common activities, more unique and expressive activities, such as dance, are cumbersome and unintuitive. Furthermore, little is known regarding the appropriate mechanical behavior required by the prosthesis during such activities. Recent improvements in bionic prosthetic devices have provided the opportunity to vary the mechanical characteristics of prosthetic joints in real-time [3,4]. These advancements have laid the foundation for prostheses that can accommodate a wide range of activity modes, including expressive activities, but the underlying control systems have yet to be studied.

The purpose of this study was to develop a control system for a bionic ankle-foot prosthesis capable of dancing the rumba, a popular Latin-American rhythm dance. Ankle joint kinematics and kinetics were obtained from a non-amputee professional dancer during various steps of the rumba. The data were used to develop a biologically inspired control system for the bionic ankle-foot prosthesis. Following testing, the ankle joint kinetics and kinematics of the bionic dance prosthesis were compared to those from a common passive prosthetic ankle worn while performing the rumba. The intent of this work was to extend the mobility of individuals with below-knee amputations to permit expressive activities that have traditionally remained difficult and unintuitive, reducing the gap in motor abilities between persons with and without leg amputation.

## Materials and Methods

### Bionic Ankle Prosthesis

**Fig 1:**
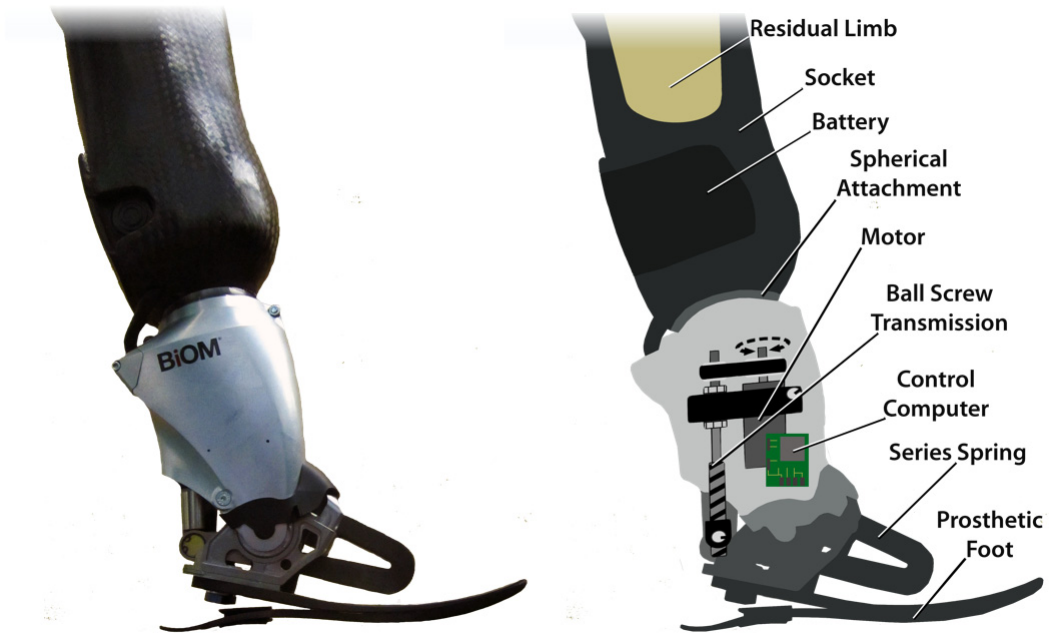
Schematic of bionic dancing prosthesis. Bionic ankle prosthesis shown (left) with major components highlighted (right). Note the location of the battery in the distal prosthetic socket.

A commercially available bionic ankle prosthesis (BiOM Inc, Medford, MA) was modified for use in the study. The bionic device was chosen for its ability to provide joint position, impedance and torque control to the wearer, as well as the ability to implement novel control programs on the computers within the device. Two physical modifications were made to the bionic ankle-foot prosthesis. The prosthesis was modified to permit a lightweight spherical attachment between the prosthesis and the prosthetic socket. This enabled the prosthetist to easily adjust the alignment of the prosthesis. Additionally the spherical mount modification permitted a space within the socket where the battery was relocated. Relocating the battery provided a more anthropomorphic shape for the prosthesis and socket, allowing the experimental participant with leg amputation to perform cross-step dance maneuvers without leg-to-leg interference. The modifications of the prosthesis reduced the total mass by 100 g (Fig 1).

### Rumba Biomechanics of a Non-Amputee

Biomechanical data were obtained from a 39 year old, female professional dancer (height: 1.75 m, mass: 54.5 kg) in the gait lab within the Biomechatronics group at the MIT Media Lab. The study was approved by the MIT Committee On the Use of Humans as Experimental Subjects and written informed consent was obtained. Five trials were obtained of four steps of the rumba, with each trial lasting approximately 5–15 seconds. The specific steps were chosen because they span a broad range of the rumba dance. The four steps included in the study are known as basic, crossovers double-rock, crossovers with underarm turn, and open break. The participant was instructed to attempt to keep one foot on the force platforms within the gait lab, if possible, to maximize the data usable for the analysis.

During the dance steps, kinetic data were collected using the force plates within an instrumented treadmill (Bertec Corporation, Columbus OH) and two in-ground force plates (AMTI, Watertown, MA). Force data were sampled at 1 kHz while the kinematic data were collected synchronously via an infrared camera system (Vicon Motion Systems, Oxford, UK) and sampled at 100 Hz. The motion capture system utilized passive markers placed at 42 (bilateral) locations on the participant's body according to the Helen Hayes marker model. The raw data were processed in SIMM (Musculographics Inc., Evanston, IL) to obtain joint trajectory and torque profiles. Data were segmented to only include regions where one foot was acting on a single force platform. The relationship between ankle torque and ankle angle was explored for the development of the controller, where negative angles denoted plantar flexion.

### Development of a Biologically-accurate Control System

The goal of the control system was to provide a biologically-accurate relationship between ankle torque and ankle angle during the rumba. The ankle torque for each step was investigated as a function of ankle angle while the angular velocity was greater than 30 degrees/s, chosen to eliminate regions of the dance steps where the ankle behavior was static. Linear regression was used to determine the mean relationship between torque and angle for each dance step. Subsequently, the slope and intercept were averaged across dance steps to obtain the mean linear torque-angle relationship that described the rumba dance steps. A linear spring-damper model of ankle mechanical characteristics was chosen for the desired behavior implemented in the control system, a model that has previously been shown to successfully characterize other human ankle behaviors [5,6]. The slope of the mean linear regression value corresponds to the stiffness and the equilibrium angle was determined by the x-intercept of the regression. In other words, the control system was defined by

$$\tau =k\left(\theta -\theta_{0}\right)+b\dot{\theta }$$ (1)

where τ corresponds to ankle torque, *k* is ankle stiffness, *θ* is ankle angle, *θ*_0_ is equilibrium angle, *b* is the damping coefficient and $\dot{\theta }$ is ankle angular velocity measured by the bionic ankle. The damping value was tuned to the preference of the amputee wearer. Following development, the control system was downloaded to the control computer within the bionic dance prosthesis (Fig 1).

### Testing with an Amputee Dancer

To quantify the performance of the biologically-inspired control system, a participant with a unilateral transtibial amputee tested the device. The participant was a 33 year old female (height: 1.72 m, weight: 54.5 kg) who lost her left leg in a traumatic amputation in April 2013. The participant is a trained, professional dancer. At the time of testing, she was 15 months post amputation and her residual limb was 40% of the length of her unaffected side (measured from the lateral epicondyle to the lateral malleolus). For daily use, she used a pin suspension with a patient adjustable Elation prosthetic foot (Össur, Reykjavik, Iceland). The participant has given written informed consent to publish these case details.

Prior to testing, the participant’s prosthetist fit her with the bionic dance leg and aligned the device. She was permitted ample time to familiarize herself with the bionic device, and the experiment proceeded only when she reported being comfortable. The control system was modified based on feedback from the participant. During testing, she completed each of the four steps of the rumba in the gait lab. Five trials of each dance step were recorded using the aforementioned experimental protocol. Following testing with the bionic dance prosthesis, she repeated the trials with her conventional prosthesis. Identical post-processing and analysis were performed, as explained above.

To quantify the biological realism of the control system, the fraction of overlapping torque-angle areas was used. For each dance step, the combined torque-angle data from all trials were segmented into areas of 3° and 0.06 Nm/kg, making a grid of torque-angle regions. The number of regions that contained data from both the non-amputee dancer and the amputee participant was divided by the total number of regions in the non-amputee data. In other words, this fraction quantified the amount of overlap between the amputee participant’s data and the non-amputee data. The fractions were calculated for both prosthesis test conditions (bionic dance leg and conventional passive prosthesis). The overlap percentages were compared with a paired, two-tailed t-test and the level of significance was set to 0.05.

## Results

### Development of a Biologically-accurate Control System

**Fig 2:**
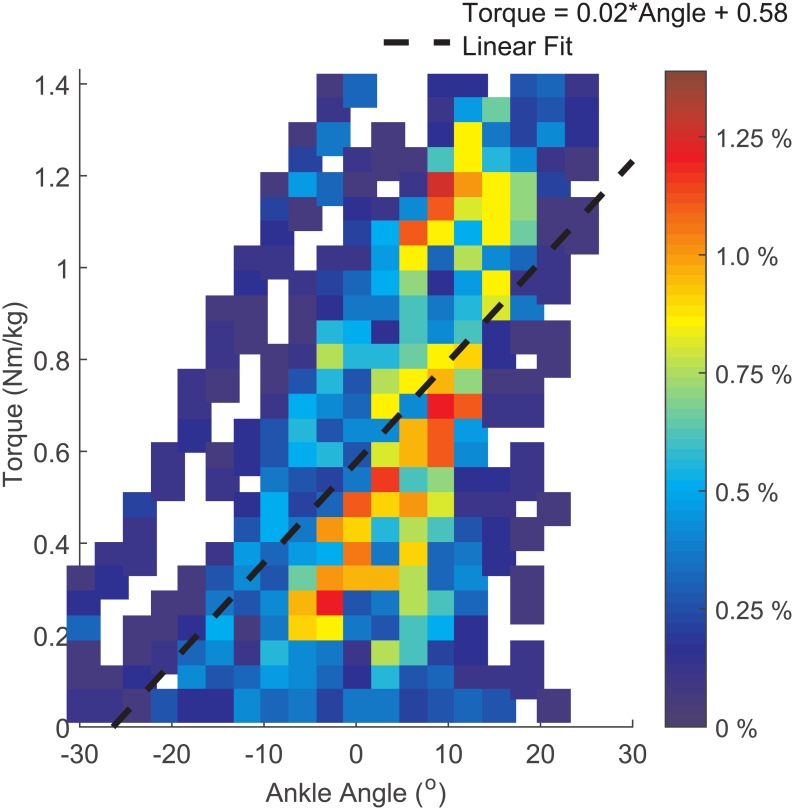
Torque vs. ankle angle data. Non-amputee ankle torque plotted against ankle angle for the under arm dance step. The color of the region indicates the percentage of the dance step data within the region; the darkest red regions had the highest concentration of data. The line of best fit is shown, which was used as the basis for the biologically inspired control system.

The way ankle torque varied with ankle angle was used as the framework for the biologically-inspired control system. The results for the linear regressions of each step are shown in Table 1 and a representative plot of the non-amputee torque-angle data is shown in Fig 2. Following feedback from the amputee participant regarding performance and comfort during the dance steps, the final values for the stiffness, equilibrium angle and damping were 0.011 Nm/°/kg, -10° and 9.7 x 10^−4^ Nms/°/kg, respectively.

**Table 1:** Regression results from non-amputee data.

Step	Slope (Nm/°/kg)	Intercept (Nm/kg)	*θ*_0_	R^2^
Basic	0.016	0.52	-32°	0.16
Double Rock	0.017	0.65	-38°	0.30
Under Arm	0.021	0.58	-27°	0.32
Open Break	0.018	0.63	-35°	0.27
Mean	0.018	0.595	-33°	

### Comparison between Bionic and Conventional Prosthesis

**Fig 3:**
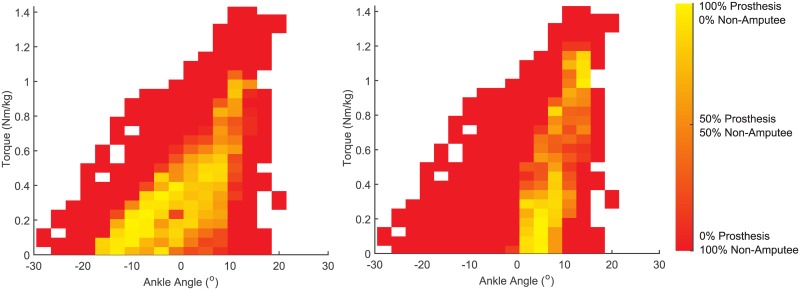
Overlap for prosthesis conditions. Ankle torque and angle data shown for the Crossovers dance step, with the bionic prosthesis condition shown on the left and the passive conventional prosthesis shown on the right. Color denotes the fraction of prosthesis data in the region compared to the non-amputee data.

The bionic control system was more biologically realistic than the passive conventional prosthesis. The passive conventional prosthesis overlapped with 26 ± 2% (mean ± standard deviation) of the non-amputee data, when averaged across the four dance steps. The bionic control system overlapped with 37 ± 6% of the non-amputee data regions (Fig 3). When compared across dance steps, the overlap of the bionic control system was statistically greater than the passive prosthesis (*p* = 0.01).

## Discussion

The bionic dance prosthesis was designed to provide the appropriate relationship between ankle angle and ankle torque, during the steps of the rumba. It should be noted that the control system was not acting in a feed-forward manner, indicating that the amputee participant was not “riding” the bionic prosthesis. Instead, she biomechanically interacted with the prosthesis through her prosthetic socket, and the biologically-appropriate kinetic and kinematic relationship was enforced.

The bionic dance prosthesis significantly outperformed the conventional prosthesis when comparing kinetics and kinematics during the rumba; however, there is much work to be done. The range of motion for current bionic dance prostheses is designed based on walking kinematics. Dancing requires a larger range of motion than walking. This motivates the need for new bionic prostheses designed specifically for such uncommon motor activities such as dance.

The control system built for the bionic device assumes a single spring-damper model for the human ankle joint. As a result, no net-positive mechanical power is added by the bionic prosthesis during the dance steps. Future work will focus on a control system that provides appropriate levels of mechanical power to ankle joint, in a comfortable, biologically appropriate way. Additionally, to be able to replicate the area shown in Fig 2 (rather than a line), the equilibrium angle would need to be varied dynamically. The stationary nature of the control system demonstrated in this work is not able to provide time-varying dynamics. Future work may focus on more specialized control systems provide time-varying behavior and dance step recognition, improving the impact of the bionic device.

This study lays the foundation for quantifying other dances and expressive activity modes, rather than only activities of daily living. By understanding such activities, devices can be created that extend the motor skills available to amputees, improving their quality of life and diminishing the gap between the abilities of amputees and non-amputees.
